# Fructose impairs brown adipogenesis by promoting thyroid hormone resistance in differentiated brown adipocytes

**DOI:** 10.1242/bio.062648

**Published:** 2026-06-22

**Authors:** Xiangdong Wu, Salaheldeen Elsaid, Lei Zhang, Eric Y. Chan, Junkai Hu, Liqing Yu, Sui Seng Tee

**Affiliations:** ^1^ Department of Diagnostic Radiology & Nuclear Medicine, University of Maryland School of Medicine, Baltimore, MD 21201, USA; ^2^ Department of Computer & Information Sciences, Towson University, Baltimore, MD 21252, USA; ^3^ Rezyko LLC, Seattle, WA 98109, USA; ^4^ Division of Endocrinology, Diabetes and Nutrition at the Department of Medicine, University of Maryland School of Medicine, Baltimore, MD 21201, USA

**Keywords:** Fructose, Brown adipocytes, Adipogenesis, Thyroid hormone resistance

## Abstract

Excessive fructose consumption is increasingly linked to obesity, diabetes, non-alcoholic fatty liver disease (NAFLD), and cardiovascular disease, yet its impact on brown adipocytes remains poorly defined. Here, we investigated how fructose alters brown adipogenesis with a focus on thyroid hormone receptor (THR) signaling. Differentiation of mouse brown preadipocytes in fructose-containing medium resulted in markedly reduced lipid droplet size and number, impaired expression of adipogenic markers (PPARγ, FABP4), and a loss of the brown adipocyte identity protein UCP-1. Whereas triiodothyronine (T3) and the THRβ-selective agonist resmetirom dose-dependently enhanced UCP-1 expression in glucose medium, neither T3 nor resmetirom restored UCP-1 in fructose medium, revealing thyroid hormone resistance. Mechanistically, fructose specifically downregulated THR but not its heterodimer partner, retinoid X receptor (RXR), via ubiquitin-mediated degradation. Overexpression of THR rescued UCP-1 in glucose medium and promoted dynamic lipid turnover but failed to restore UCP-1 or lipid remodeling in fructose medium, indicating a defect in THR transcriptional regulation. Our secretome proteomics revealed that both the sugar source and THR overexpression strongly remodel the secreted protein landscape of brown adipocytes. The data indicate that fructose both reconfigures metabolic and signaling networks and blunts oxidative resilience, while excess THR activity in fructose conditions disrupts energy metabolism and increases the risk of protein misfolding. All these could contribute to THR dysfunction and impair adipogenesis. Collectively, these findings identify fructose as a potent inhibitor of brown adipogenesis, suppressing THR-dependent UCP-1 expression by inducing thyroid hormone resistance through a defect in THR-mediated transcriptional regulation. This mechanism links dietary fructose to impaired brown adipocytes and metabolic imbalance.

## Teaser

**Summary:** Our findings reveal a previously unrecognized mechanism whereby fructose induces thyroid hormone resistance, suppresses UCP-1 expression, and impairs brown adipocyte differentiation and thermogenic programming.

## INTRODUCTION

The risk of metabolic diseases such as obesity, diabetes, non-alcoholic fatty liver disease (NAFLD), and cardiovascular diseases related to fructose is rising at an alarming level worldwide (Wang et al., 2022; Pang et al., 2020; Jensen et al., 2018; Chen et al., 2020; Stanhope, 2016). Emerging evidence suggests that brown adipocytes play a crucial role in the effects of fructose on metabolic homeostasis, particularly when fructose intake increases and blood fructose levels rise (Razzoli et al., 2016; Neto et al., 2022; Liu et al., 2023; Tulp, 2023). With higher expressing transporters (GLUT-2, GLUT-5), a crucial metabolic enzyme, ketohexokinase (KHK) (Trio Pamungkas et al., 2023; De Vito et al., 2024; Hussar et al., 2016; Damen et al., 2021; Wali et al., 2023), and direct blood inflow from the portal vein, the liver is considered a primary metabolic hub for fructose under normal conditions. However, as blood levels increase, fructose also reaches peripheral tissues, including brown adipose tissue (BAT). Brown adipocytes express the fructose transporter GLUT-5 and the metabolic enzyme KHK-C in an inducible manner (Reinisch et al., 2022; Diggle et al., 2009; Elsaid et al., 2024). Therefore, brown adipocytes are mechanically ready to uptake and metabolize fructose. Due to their high lipid and glucose consumption for thermogenesis, brown adipocytes play a critical role in maintaining energy and metabolic homeostasis (Cypess, 2022), especially when fructose disrupts normal glucose and lipid metabolic processes. However, the impact of fructose on brown adipocytes and their thermogenic potential remains poorly defined. Thyroid hormone receptors (THRs) are nuclear receptors that can directly bind to the thyroid hormone response element (TRE) in the promoter region of UCP-1, thereby directly regulating the transcription of UCP-1 (Sentis et al., 2021; Lee et al., 2012; Endo and Kobayashi, 2008). Here, we investigated whether fructose alters the differentiation of brown preadipocytes into mature adipocytes, specifically by examining UCP-1 expression. This is a crucial step in exploring the mechanism by which fructose affects brown adipocyte function and its metabolic consequences.

## RESULTS

### Fructose reduced the number and size of cellular lipid droplets and retarded adipogenesis in morphology, but had no significant impact on the quantity of total protein and DNA

**Fig. 1.**
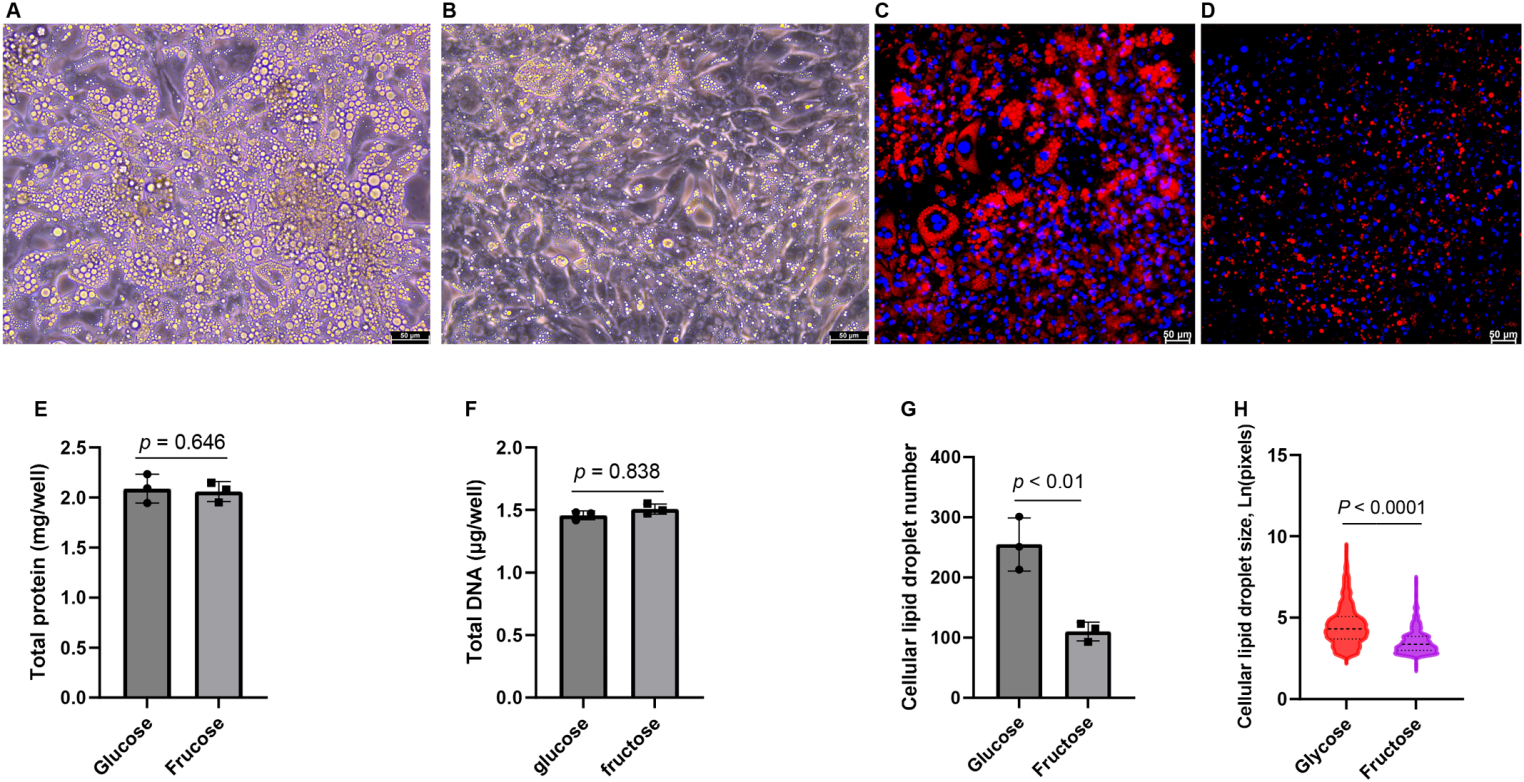
**Lipid, protein, and DNA content in differentiated brown adipocytes cultured in the medium including different hexoses (glucose versus fructose).** Cell images were acquired using a Leica DMi8 microscope with visible light in phase-contrast or fluorescence mode. The wavelength of fluorescence excitation/emission is 637/655 nm for lipid TOX deep red neutral lipid stain and 460/360 nm for Hoechst 33342 nuclei stain. Images were processed by Leica Application Suite X software. The magnification was 10×20. (A) Cells differentiated in glucose-containing medium with phase contrast image; (B) cells differentiated in fructose-containing medium with phase contrast image; (C) lipid droplets staining for the cells differentiated in glucose-containing medium with fluorescence image; (D) lipid droplets staining for the cells differentiated in fructose-containing medium with fluorescence image. (E) Total protein in each well of the six-well plate (*n*=3). (F) Total DNA in each well of the six-well plate (*n*=3). (G) Cellular lipid droplet number quantified from three images/three wells, (*n*=3). (H) The distribution of cellular lipid droplet size (violin plot) with all lipid droplets of three images from each group. Data were presented as mean±s.e.m. for panels E, F, and G.

The effects of fructose on adipocytes, especially on brown adipocytes, are not well characterized. To explore the impact of fructose on brown adipocytes, we first cultured mouse brown preadipocytes in a glucose-containing medium. Once the cells reached 100% confluence, we induced and differentiated them in media containing different hexoses, specifically glucose versus fructose. The cells in glucose-containing medium had large, round, and multiple lipid droplets, a typical morphology of mature brown adipocytes. However, the size and number of lipid droplets in cells cultured in fructose medium were significantly reduced, with no disruption of total protein or DNA levels (Fig. 1). The cells lacked fully differentiated brown adipocyte morphology. These data indicate fructose has a dramatic impact on lipid metabolism and obstructs brown adipogenesis at least at the morphological level.

### Fructose obstructs brown preadipocytes from fully differentiating into mature brown adipocytes

**Fig. 2.**
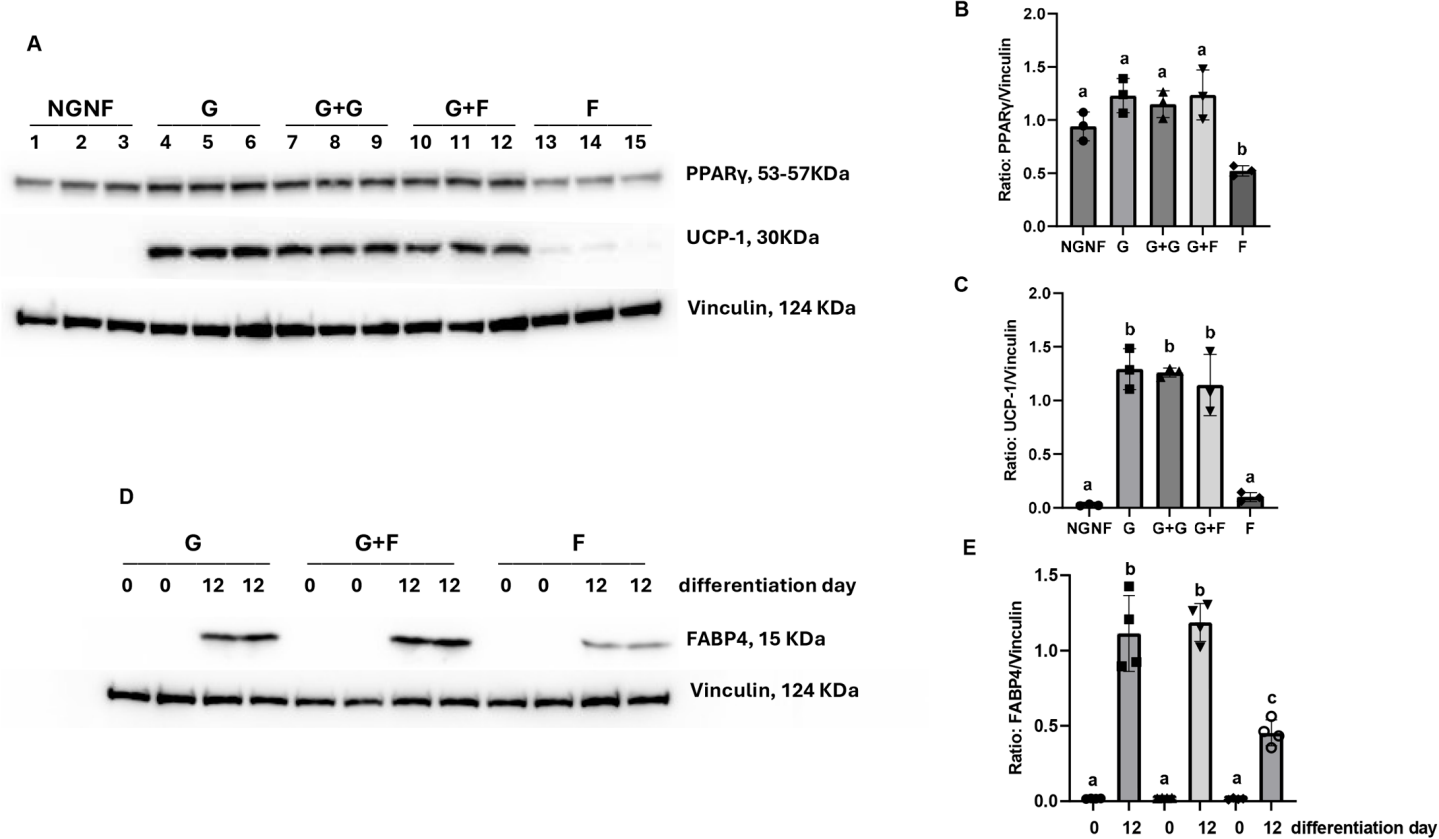
**Adipogenesis markers, PPARγ, FABP4, and brown adipocyte marker UCP-1 expression in differentiated brown adipocytes.** Brown preadipocytes were induced and differentiated in media containing different combinations of glucose and fructose. Cells were frozen and lysed on differentiation day 0 or day 12. Protein expressions of PPARγ (A,B), UCP-1 (A,C), and FABP4 (D,E) were assayed by standard western blot. (A,D) Representative western blot of PPARγ, UCP-1, FABP4. Western blots were quantified using ImageJ. Columns labeled with different lowercase letters are statistically different by ANOVA. Data were presented as mean±s.e.m., *P*<0.05, *n*=3 (PPARγ, UCP-1), *n*=4 (FABP4). G, glucose; F, fructose.

Our cell image data show that fructose significantly reduces the size and number of lipid droplets in differentiated brown adipocytes and blocks brown adipogenesis, as assessed by morphological analysis. We further investigated the expression of key brown adipogenesis markers, including PPARγ, FABP4, and UCP-1. Our data indicated that the brown adipogenesis markers PPARγ, FABP4, and UCP-1 were significantly reduced in cells induced and differentiated in a medium containing only fructose, suggesting that fructose obstructs brown preadipocytes from fully differentiating into mature brown adipocytes. However, the brown adipogenesis markers were not significantly changed in the medium mixed with fructose and glucose compared to the glucose-only-containing medium, suggesting that brown differentiated adipocytes preferentially take glucose as an energy source (Fig. 2).

### THR ligand T3 or the THR-beta agonist resmetirom did not rescue the loss of UCP-1 expression in a fructose environment, indicating thyroid hormone resistance

**Fig. 3.**
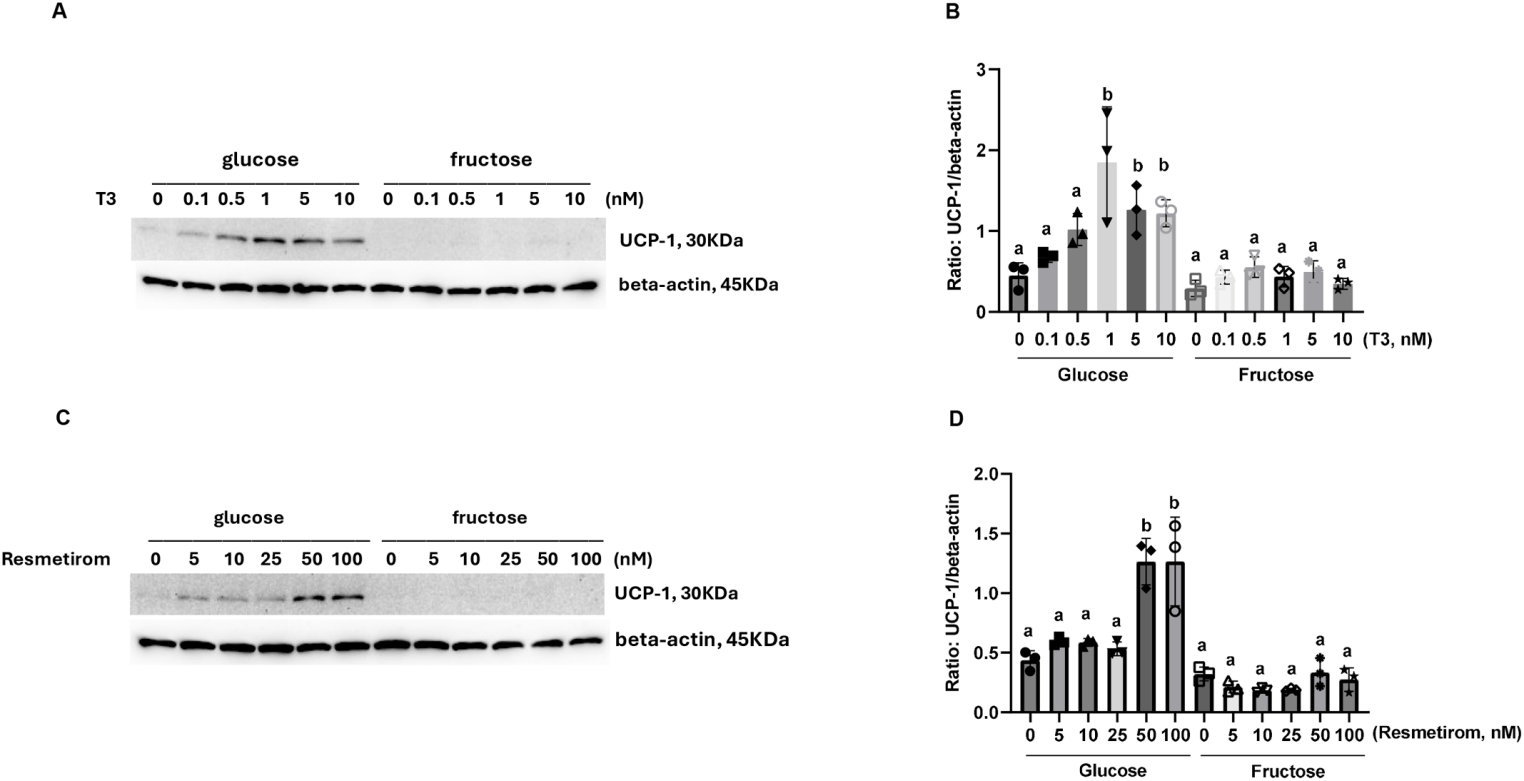
**UCP-1 protein expression with different concentrations of THR ligand triiodothyronine (T3) and THR-beta agonist resmetirom (Rezdiffra).** Brown preadipocytes were induced and differentiated in media containing different concentrations of T3 (A,B) or resmetirom (C,D). Cells were frozen and lysed on day 12 of differentiation. Protein expressions of UCP-1 were assayed by standard western blot. (A,C) Representative western blot of UCP-1. Western blots were quantified using ImageJ (B,D). Columns labeled with different lowercase letters are statistically different by ANOVA. Data were presented as mean±s.e.m., *n*=3, *P*<0.05.

Thermogenic protein UCP-1 is an identity protein marker of mature brown adipocytes. UCP-1 is a THR direct transcriptional target. It is significantly reduced in differentiated brown adipocytes induced and differentiated in a medium containing only fructose. To test whether increasing THR ligand T3 or the THR-beta agonist resmetirom can restore UCP-1 expression in a fructose-rich environment, we induced and differentiated brown preadipocytes in medium supplemented with varying concentrations of T3 and resmetirom. We harvest cells on day 12 of differentiation and perform a western blot. When cells were cultured in glucose-containing medium, as expected, UCP-1 protein expression increased with increasing T3 or resmetirom concentration. UCP-1 expression reached a peak level as T3 concentration reached 1 nM, and then slightly decreased when T3 concentration further increased to 5 or 10 nM. UCP-1 expression levels peak at 50 nM resmetirom and remain at this level as the concentration increases to 100 nM. However, when the cells were cultured in a fructose-only medium, UCP-1 expression was very low and did not increase with increasing concentrations of T3 and resmetirom. Our data suggest that the thyroid hormone receptor ligand T3 and the THR-beta agonist resmetirom promote UCP-1 protein expression in a dose-dependent manner in a glucose environment. However, T3 and resmetirom did not restore the loss of UCP-1 expression observed in a fructose-rich environment (Fig. 3). These data suggests that thyroid hormone resistance in UCP-1 expression occurs when brown adipocytes are cultured in a fructose-rich medium.

### Only one binding partner, THR, was negatively altered in the THR-RXR heterodimers in the fructose-rich culture environment

**Fig. 4.**
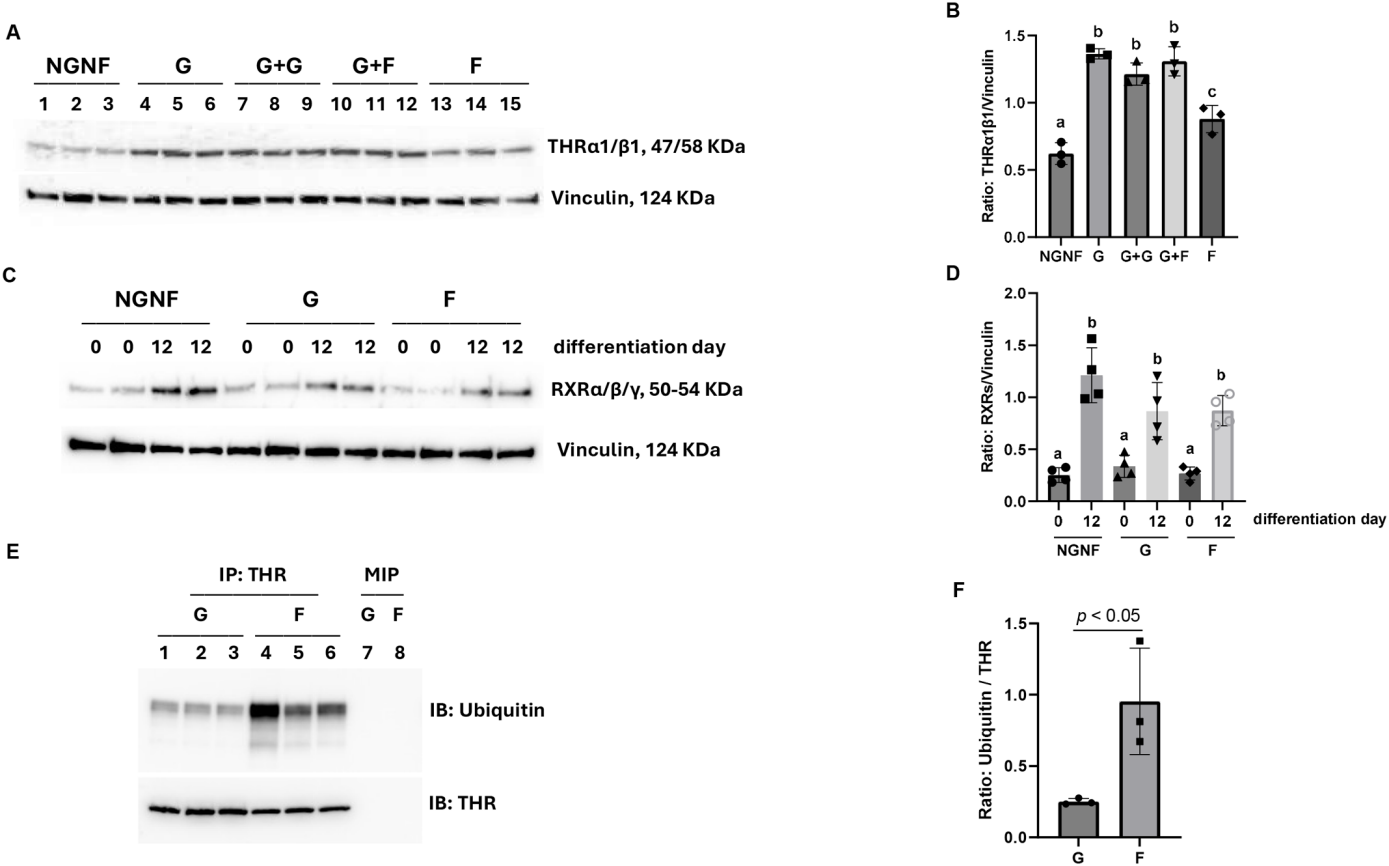
**THRα1β1 and RXRα/β/γ expression and ubiquitination of THRα1β1 in differentiated brown adipocytes.** Brown preadipocytes were induced and differentiated in media containing various combinations of glucose and fructose. Cells were frozen and lysed on differentiation day 0 or day 12. Protein expressions of THRα1β1 (A,B) and RXRα/β/γ (C,D) were assayed by standard western blot. (A,C) Representative western blot of THRα1β1 and RXRα/β/γ. Ubiquitination of THRα1β1 (E) was assayed by co-immunoprecipitation. THRs were immunoprecipitated from cell lysate and immunoblotted with an ubiquitin antibody. In panels B and D, columns labeled with different lowercase letters are statistically different by ANOVA. Data were presented as mean±s.e.m., *P*<0.05, *n*=3 (THRα1β1, ubiquitin), *n*=4 (RXRα/β/ϒ). NGNF, no glucose and no fructose; G, glucose; F, fructose; IP, immunoprecipitation; MIP, mock immunoprecipitation.

The thyroid hormone receptors (THRα1β1) required retinoid X receptors (RXRα/β/γ) as a binding partner to form a heterodimer to stably bind to thyroid hormone response elements (TREs) in the promoter region of the target gene UCP-1 (Endo and Kobayashi, 2008; Zhang and Lazar, 2000). This step is crucial for the function of THR transcription regulation. To test if fructose alters THRα1β1 or RXRα/β/γ expression, we induced and differentiated brown preadipocytes in the medium that contained various combinations of glucose and fructose. We then froze and lysed cells on differentiation day 12, followed by western blot analysis to assess protein expression (Fig. 4). Our results show that THR-expression was significantly reduced in cells cultured in fructose-only medium. However, the expression of retinoid X receptors did not differ significantly between cells grown in glucose- or fructose-containing medium. This result suggests that only one heterodimer partner, THR, was downregulated in a fructose-only medium.

### Post-translational modification ubiquitination is involved in the degradation of the THR in a fructose-rich environment

THR expression is regulated by a multi-layered set of mechanisms, including transcriptional, post-transcriptional, hormonal, and metabolic regulation, as well as post-translational modifications (Kim et al., 2021; Ortiga-Carvalho et al., 2014). Given that the cells were cultured in a differential-hexose environment, we first explored possible post-translational regulation of THRs. To test whether fructose alters the ubiquitination of THR (α1β1), we first immunoprecipitated THRs from cell lysates and then immunoblotted them with a ubiquitin antibody to assess their ubiquitination status (Fig. 4). Our results indicated that the ubiquitination of THRs was significantly increased in cells induced and differentiated in fructose medium, suggesting that the ubiquitin-proteasome system (UPS) was involved in THR degradation.

### Resuming THR expression did not rescue UCP-1 expression in a fructose-rich environment

**Fig. 5.**
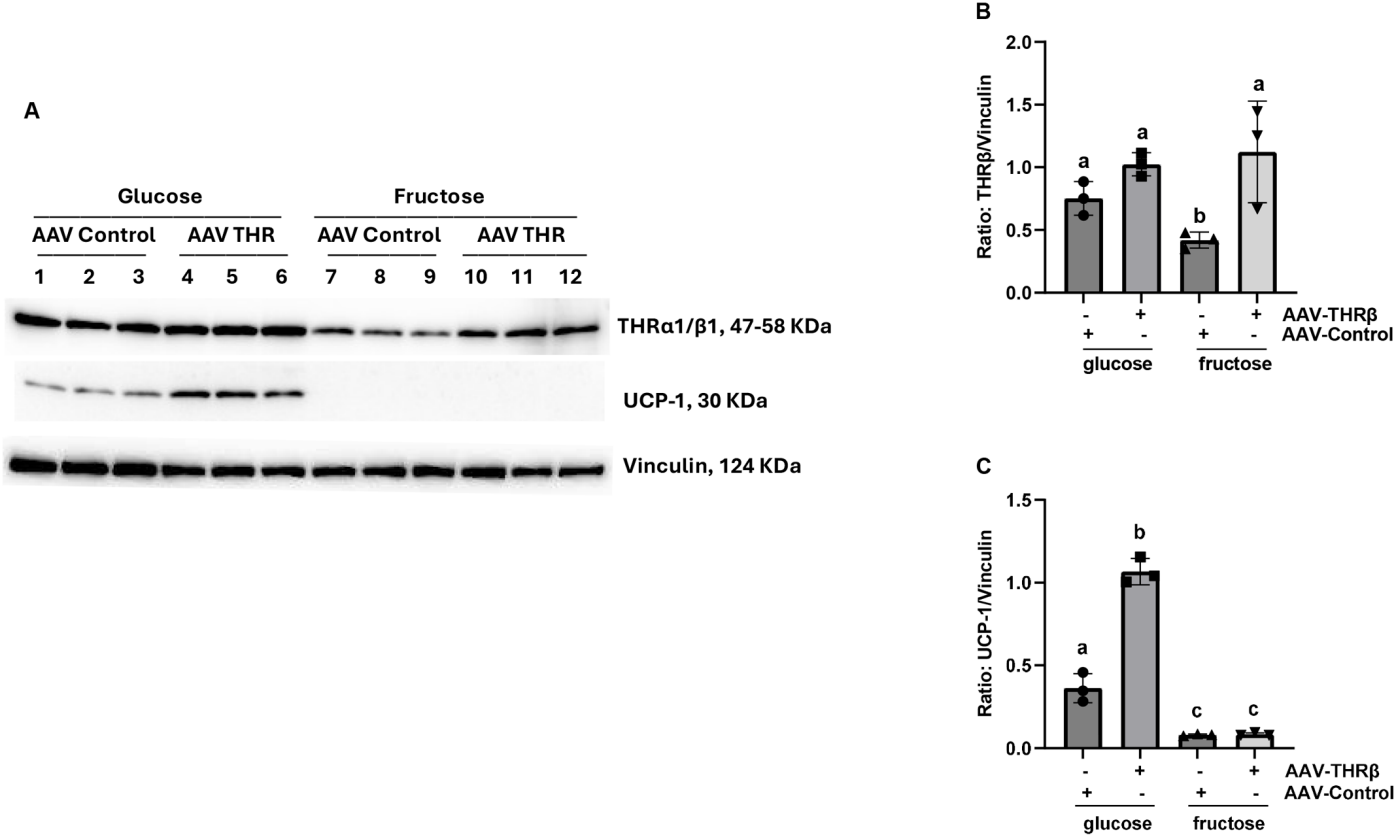
**Protein expression of THRα1β1 and UCP-1 in differentiated brown adipocytes at Day 12.** Cells were differentiated in glucose or fructose-containing medium. Cells were also transduced with an AAV containing an EGFP plasmid as a control or a thyroid hormone receptor-β plasmid to overexpress THR. Protein expressions of THRβ (A,B) and UCP-1 (A,C) were assayed by western blot. (A) Representative western blot of THRα1β1 and UCP-1. Western blots were quantified using ImageJ (B,C). Columns labeled with different lowercase letters are statistically different by ANOVA. Data were presented as mean±s.e.m., *P*<0.05, *n*=3.

Fructose downregulated THR and suppressed UCP-1 expression. To test whether resuming THR expression in cells cultured in fructose-containing medium could restore UCP-1 expression, we used AAV-mediated transduction to overexpress THR and examined protein levels of both THR and UCP-1 at day 12 of differentiation. In cells cultured in glucose medium, as expected, UCP-1 levels increased with increasing THR expression. However, UCP-1 expression remained low despite elevated THR expression, similar to that observed in glucose conditions (Fig. 5). This result indicates a disconnect between THR and UCP-1 expression in cells cultured in a fructose-containing medium. This disconnection also suggests a defect in the transcriptional regulation of THR, leading to thyroid hormone resistance.

### Overexpressing THR in a glucose-rich environment reduced the size of cellular lipid droplets, whereas resuming THR expression in a fructose-rich environment did not significantly alter their number or size

**Fig. 6.**
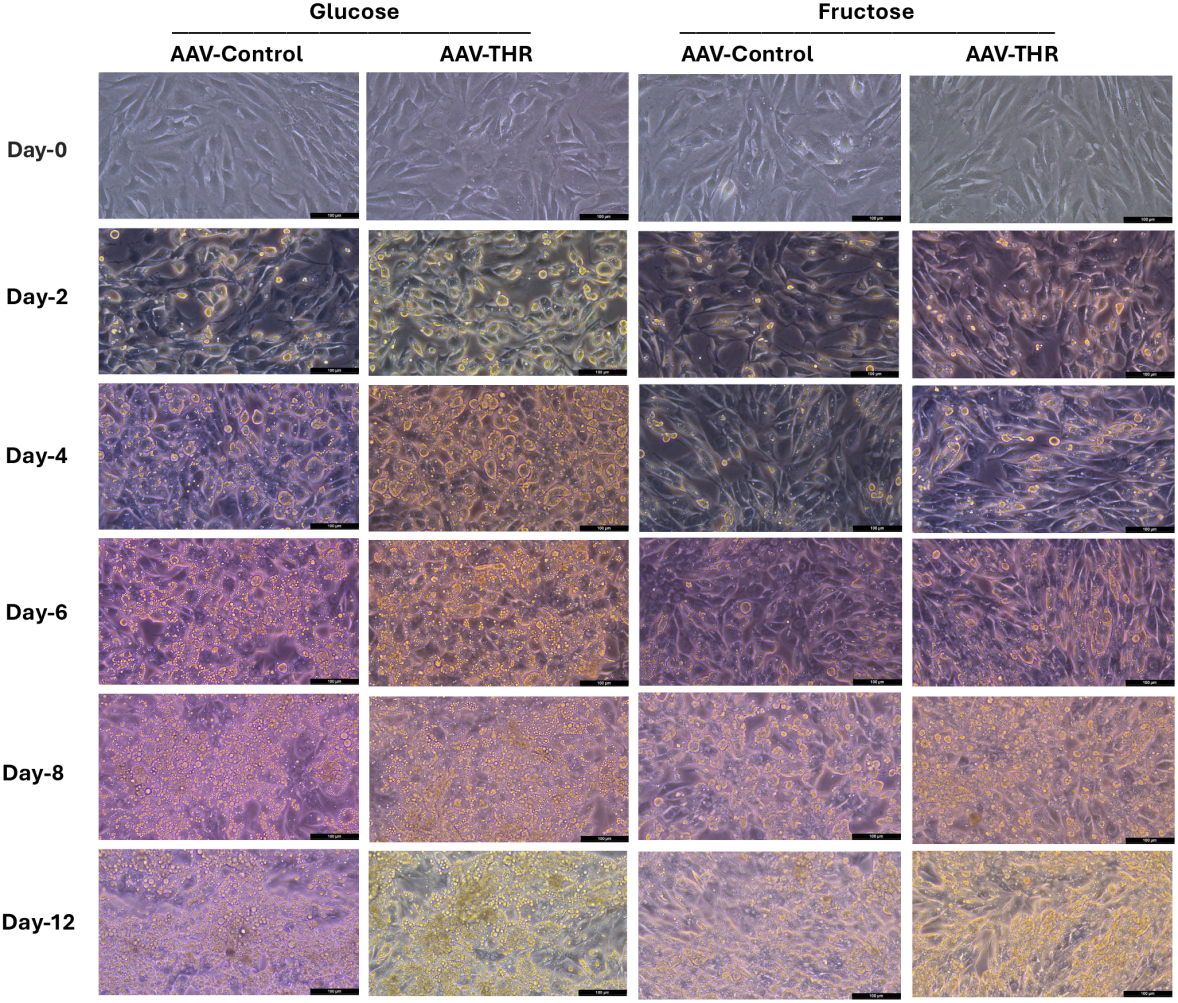
**Morphology of immortalized brown preadipocyte and differentiated brown adipocyte during 12-day differentiation.** The stromal vascular fraction (SVF) cells were isolated from the interscapular brown adipose tissue (iBAT) of 1-day-old mouse pups. Brown preadipocytes were immortalized by expression of the large T antigen. Cells were induced and differentiated in induction medium and differentiation medium for 12 days. Cell images were acquired using a Leica DMi8 microscope with visible-light, color-channel, and phase-contrast settings. Images were processed by Leica Application Suite X software. The magnification was 10×20.

**Fig. 7.**
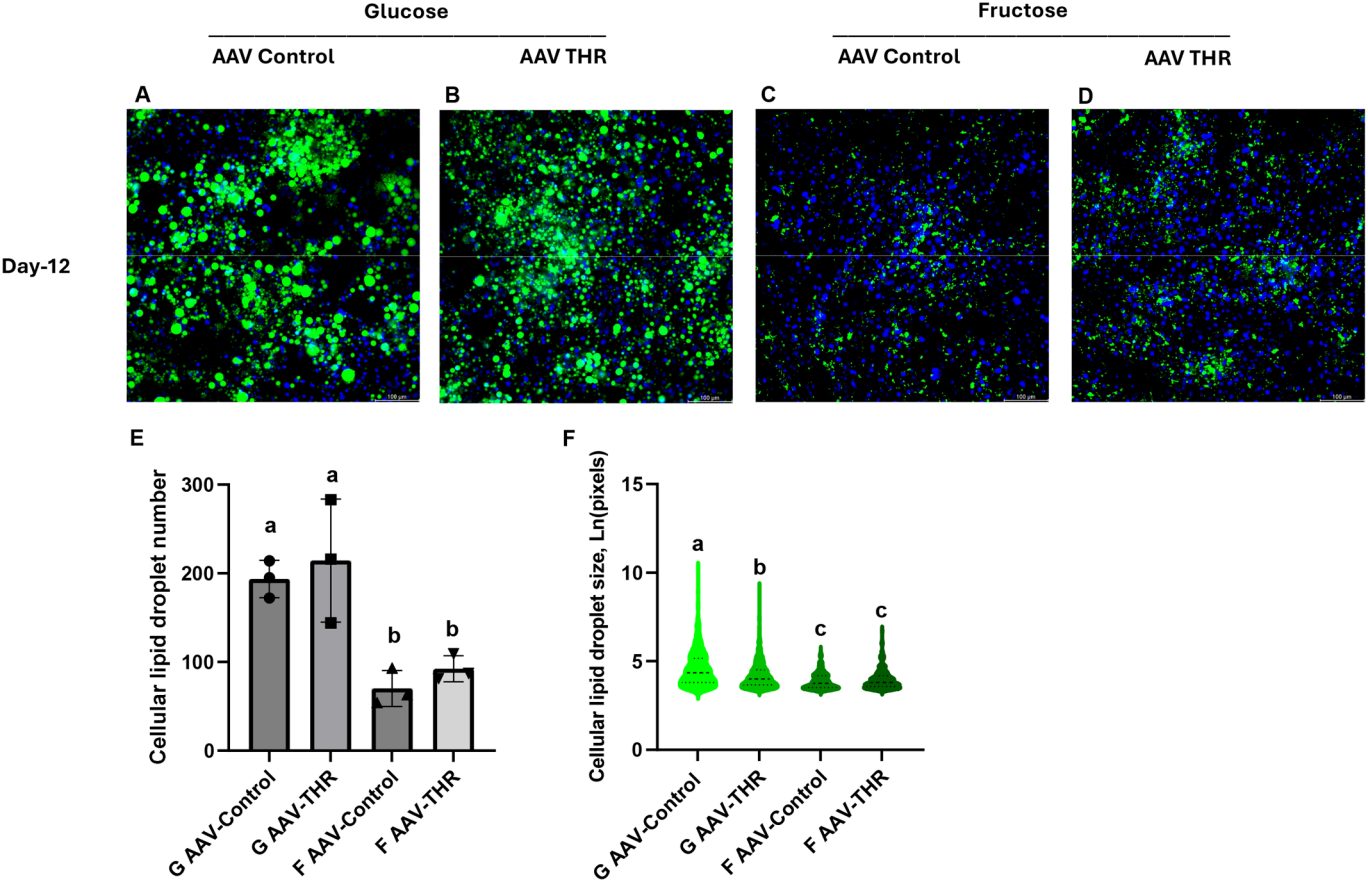
**Fluorescence image of lipid droplets and nuclei in the cells on day 12 of differentiation.** Cells in differentiation day 12 were stained with BODIPY green neutral lipid stain and Hoechst 33342, respectively. Cell images were acquired using Leica DMi8 microscopy with fluorescence settings. The wavelength of fluorescence excitation/emission is 493/503 nm for BODIPY green neutral lipid stain and 460/360 nm for Hoechst 33342 nuclei stain. Images were processed by Leica Application Suite X software. The magnification was 10×20. The lipid droplets and nuclei staining in the cells under the conditions of (A) glucose+AAV-control; (B) glucose+AAV-THR; (C) fructose+AAV-control; (D) fructose+AAV-THR. (E) Cellular lipid droplet number was quantified from three images/three wells (*n*=3); data were presented as mean±s.e.m. (F) The distribution of cellular lipid droplet size (violin plot) with all lipid droplets of three images from each group. Columns labeled with different lowercase letters are statistically different by ANOVA, *P*<0.05.

Fructose downregulated THR, while also decreasing the number and size of cellular lipid droplets. To evaluate the impact of elevating THR expression on cellular lipid droplets, we overexpressed THR using AAV-mediated transduction in cells cultured in glucose- or fructose-containing medium. As expected, overexpression of THR significantly reduced the size of cellular lipid droplets in cells cultured in glucose-containing medium, indicating a high rate of lipid turnover. Meanwhile, restored THR expression in cells cultured in a fructose-containing medium did not significantly alter the number or size of cellular lipid droplets, suggesting low lipid turnover (Figs 6,7).

### The secretome of differentiated brown adipocytes

**Fig. 8.**
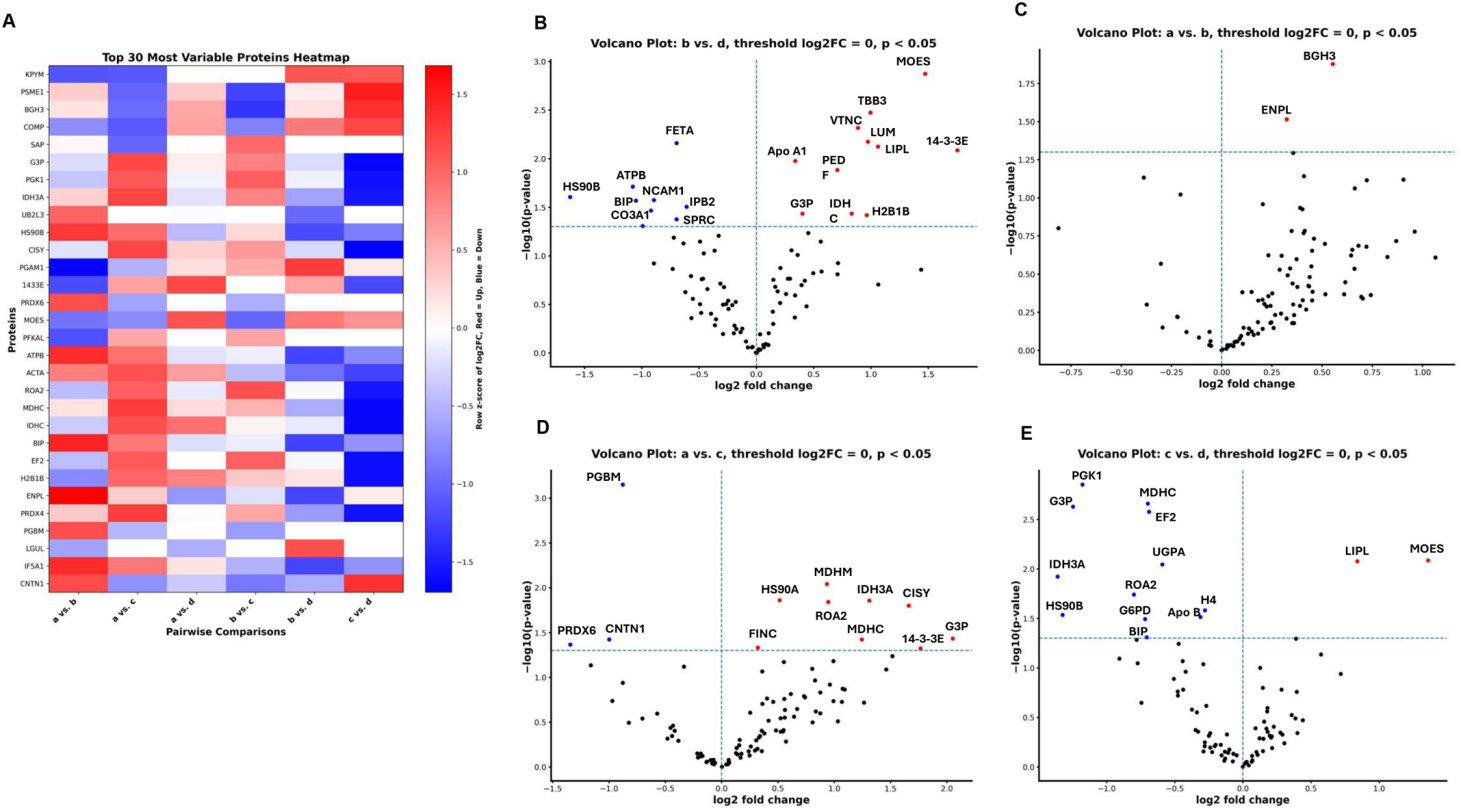
**The quantified secretomes of differentiated brown adipocytes.** Cell culture media were collected on day 12 of brown adipocyte differentiation, and the secretome was analyzed by LC-MS. (A) Heatmap of differentially regulated proteins between the groups as determined by a two-sample *t*-test. Four different treated groups were included: (a) AAV-control+glucose; (b) AAV-THR+glucose; (c) AAV-control+fructose; (d) AAV-THR+fructose. Volcano plot depicting a two-sample *t*-test between all quantified proteins in differentiated brown adipocytes with (B) AAV-THR+glucose versus AAV-THR+fructose; (C) AAV-control+glucose versus AAV-THR+glucose; (D) AAV-control+glucose versus AAV-control+fructose; (E) AAV-control+fructose versus AAV-THR+fructose. Red, upregulated; Blue, downregulated. Each group, *n*=3.

To explore how sugar source (glucose versus fructose) or THR overexpression alters the secretomes of brown adipocytes, we collected cell culture media and performed proteomic analysis using high-resolution MS. Our data (Fig. 8) indicate that sugar source (glucose versus fructose) dramatically alters the secretomes of brown adipocytes. Fructose significantly increases the levels of proteins involved in glycolysis and the TCA cycle/malate-aspartate shuttle, suggesting that limiting the carbon source will feedback-regulate energy metabolism. Fructose also increased the levels of proteins associated with mRNA processing/RNA binding, protein folding, and 14-3-3E-mediated cell signaling, suggesting that it serves as a signaling molecule and plays regulatory roles in many cellular processes. Fructose reduced proteins related to cell adhesion and cell redox regulation, such as peroxiredoxin 6 (Prdx6), which diminishes hydrogen peroxide and organic hydroperoxides to protect against oxidative stress. This result supports our hypothesis that fructose impairs the THR signal by oxidatively modifying the zinc-finger motif in its binding domain. In fructose-containing medium, the lipid droplets in cells were already small (Fig. 7). It was barely possible to observe any difference between THR overexpression and without THR overexpression. However, overexpression of THR significantly altered the cells' secretome. Overexpression of THR significantly reduces proteins involved in glycolysis, the TCA cycle, mRNA processing, and protein chaperones (HSP90B, BIP, etc.), which help stabilize and properly fold proteins or degrade them. This suggests that, in fructose carbon access, overdriving THR actually reduces the proteins involved in glycolysis-related energy metabolism and increases the risk of protein misfolding.

## DISCUSSION

Our data demonstrated that fructose reduced the number and size of cellular lipid droplets and that fewer preadipocytes committed to fully differentiating into mature brown adipocytes. The cells also lack the mid-stage differentiation marker PPARγ, the late-stage maturation marker FABP4, and the brown adipocyte identity marker UCP-1. Together, these data demonstrate that fructose impairs brown adipogenesis, thereby negatively impacting brown adipocyte mass and function and leading to future detrimental consequences for whole-body energy balance and metabolic homeostasis.

The THRs heterodimerize with the retinoid X receptor (RXR). The THR/RXR complex then binds to TREs in the promoter regions of target genes such as UCP-1 (Endo and Kobayashi, 2008; Mulero et al., 2013; Barbe et al., 2001; Fukuda et al., 2007). Without T3 ligand, the THR/RXR complex recruits corepressors, providing docking sites for histone deacetylases (HDACs), which remove the acetyl group from histone. This histone deacetylation results in a more compact chromatin structure, rendering transcriptional factors, RNA polymerase, and other proteins inaccessible to DNA and thereby preventing gene expression (Freudenthal et al., 2019; Fozzatti et al., 2011; Fozzatti et al., 2013; Astapova and Hollenberg, 2013). Once bound to ligand T3 or its agonist, the THR conformation changes, leading to the detachment of co-repressors and the recruitment of co-activators, which retain histone acetyltransferase (HAT) activity. HAT then adds acetyl groups to histone proteins. The acetylation of histones loosens chromatin structure, making the transcriptional machinery more accessible to DNA and enabling transcription initiation (Fozzatti et al., 2011, 2013).

Ligand binding to THR is a crucial step for transcriptional regulation of gene expression. Our data indicate that increasing ligand T3 or resmetirom did elevate THR transcriptional target UCP-1 expression in a glucose-rich environment. However, as the THR ligand T3 or resmetirom increased, UCP-1 expression did not increase and remained at a lower level in a fructose-rich environment, suggesting a defect in THR transcriptional regulation. The defect could be in the binding between the ligand and THR, in THR transformation, in THR/RXR complex formation, in the binding between the THR/RXR complex and DNA (TREs), or in the swap of co-repressor and co-activator. This defect leads to thyroid hormone resistance, which reduces UCP-1 expression. Future investigation is needed to explore the defect.

The THR and RXR are nuclear receptors that bind to specific DNA sequences and regulate gene expression in response to their ligands. Besides ligand T3, the expression level of THR and its heterodimer partner RXR are crucial for this transcriptional regulation. Our data show that only the single binding partner THR was downregulated in a fructose-rich environment. A low level of THR expression will weaken the THR/RXR formation and negatively impact THR transcriptional regulation. THRs are regulated by multilayer mechanisms (Nagy et al., 1999; Moore and Guy, 2005; Yen, 2001; Tarim, 2011; Richter et al., 2011). Cells cultured in different hexose environments (glucose versus fructose) are more relevant to cells in various nutritional statuses and stress signals. Within this context, post-translational modifications (PTMs) are likely to play a more significant role in THR turnover. Indeed, our data show that fructose increased THR ubiquitination, suggesting that post-translational modification ubiquitination is involved in the degradation of the THR in a fructose-rich environment.

**Fig. 9.**
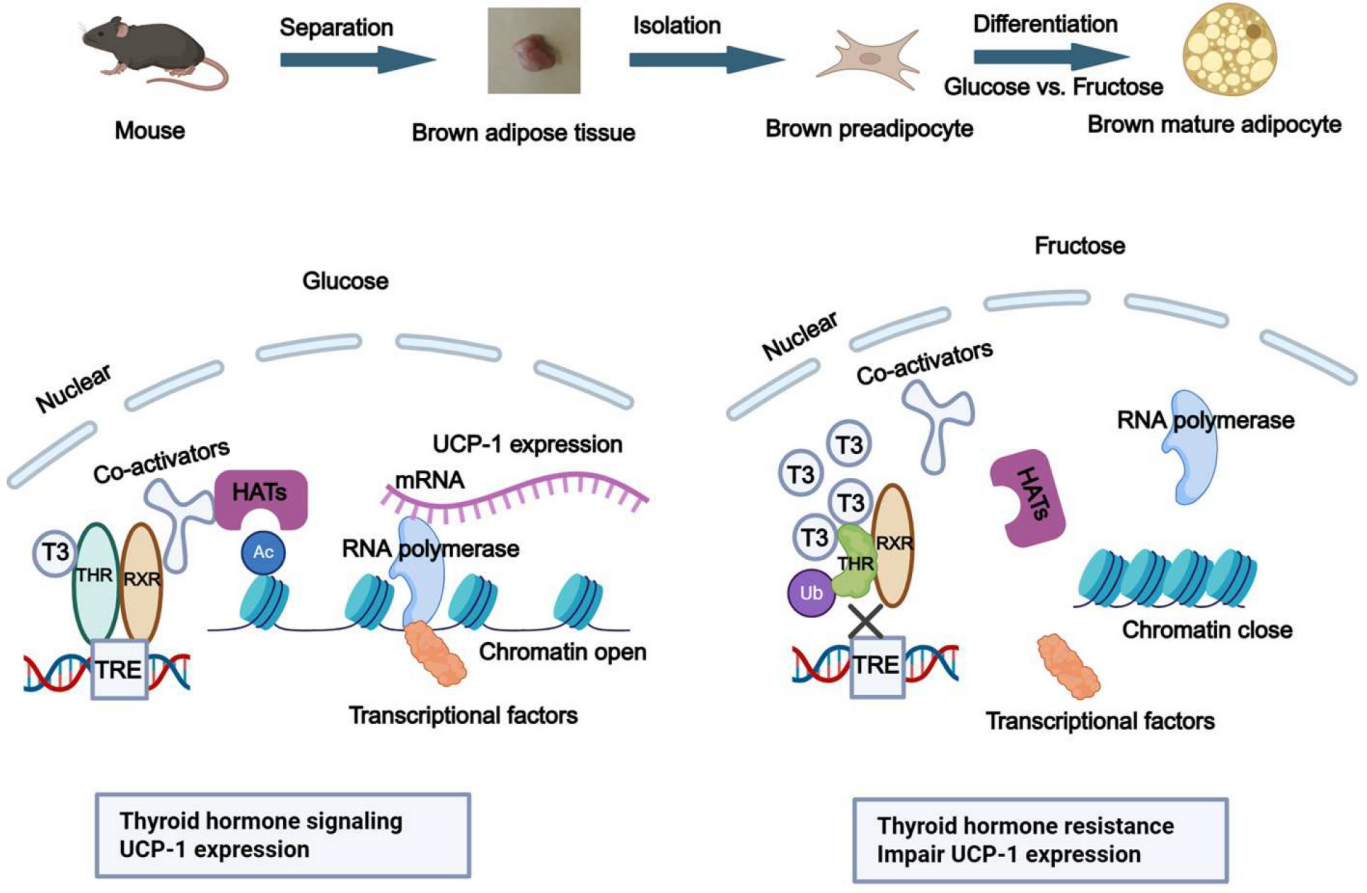
**Proposed model: glucose–fructose divergence in THR-dependent regulation of UCP-1 transcription in brown adipocytes.** Under glucose conditions, thyroid hormone (T3) binding induces a conformational change in the THR/RXR heterodimer, enabling high-affinity binding to TREs within the UCP-1 promoter. Ligand-activated THR/RXR recruits transcriptional co-activators that either possess intrinsic HAT activity or serve as scaffold proteins to recruit and stabilize HAT-containing complexes. Localized histone acetylation relaxes chromatin architecture, promotes nucleosome displacement, and facilitates ordered assembly of transcription factors, RNA polymerases, and associated co-factors, thereby driving robust UCP-1 transcription. In contrast, fructose exposure enhances THR ubiquitination, thereby accelerating proteasomal degradation. The post-translational modifications (PTMs) also induce conformational distortion of the receptor. These events directly disrupt THR interaction with TREs and destabilize the THR/RXR transcriptional complex, leading to failure of co-activator and HAT recruitment. The consequent absence of promoter-localized histone acetylation enforces a transcriptionally repressive chromatin state, precluding productive assembly of the transcriptional machinery and effectively silencing UCP-1 gene expression. The defect in the interaction between THR and TRE disrupts thyroid hormone signaling, leading to thyroid hormone resistance.

This leads to the future question. Could resume THR expression rescue UCP-1 expression in a fructose-rich environment? According to our data, overexpressing THR increased UCP-1 expression in a glucose-rich environment; however, resuming THR expression did not rescue UCP-1 expression in a fructose-rich environment. This discrepancy between THR regulation and UCP-1 expression indicates that a defect in THR transcriptional regulation occurs in a fructose-rich environment. This result also provides evidence that fructose causes thyroid hormone resistance and reduces UCP-1 expression. It is reported that mutations in the ligand-binding domain of THRβ lead to thyroid hormone resistance (Kalikiri et al., 2017; Lai and Chen, 2022; Ortiga-Carvalho et al., 2014; Yao et al., 2019; Cannarella et al., 2022; Guo et al., 2016). In the context of changes in the nutritional environment, we speculated that fructose-induced thyroid hormone resistance was not mediated by mutations in the ligand-binding domain. Our proteomics analysis of the differentiated brown adipocyte secretome indicated that fructose markedly reduced the secretion of antioxidant proteins, such as Prdx6, suggesting an increased oxidative burden. The THR DNA-binding domain contains redox-sensitive cysteine residues within its zinc-finger motifs, which are known to be susceptible to oxidative modification in other nuclear receptors (Carter and Ragsdale, 2014; Okamoto et al., 1999; Whittal et al., 2000; Maynard and Covell, 2001; Maret, 2006; Erdős et al., 2018). Based on these two observations, we hypothesized that fructose alters the zinc finger motif in the THR-binding domain, preventing THR from binding to the TRE in the UCP-1 promoter. This will result in thyroid hormone resistance to the regulation of UCP-1. Fig. 9 illustrates the proposed mechanism by which fructose impairs thyroid hormone signaling, thereby disrupting THR-dependent transcriptional regulation of UCP-1. Future investigation is needed to test this hypothesis.

THRs affect a wide range of tissues and metabolic processes. THRs directly transcriptionally regulate genes related to thermogenesis (UCP-1) (Endo and Kobayashi, 2008; Lee et al., 2012) and fatty acid oxidation (CPT1α, MCAD, ACOX1) (Zinke et al., 2003; Cheng et al., 2010; Zhu and Cheng, 2010; Zhang et al., 2021). THRs also indirectly regulate lipolytic genes (ATGL, HSL, MGL, LPL) (Ogasawara et al., 2012; Roy et al., 2017; Kershaw et al., 2007; Smih et al., 2002; Mariash, 2003; Kersten, 2014) via the PPAR pathway or adrenergic signaling crosstalk. Overall, THRs promote energy expenditure.

THRs also regulate genes related to lipogenesis, such as PPARs, ACC, FASN, SCD1, and DGAT (Araki et al., 2005; Adams et al., 2010; Thakran et al., 2013; Levacher et al., 1988; Hashimoto et al., 2006; Olichwier et al., 2020; Mendoza et al., 2021). Currently, there is insufficient data to support the presence of a validated TRE in the promoter region of these genes, which is necessary for binding by the THR/RXR heterodimer. Therefore, THRs can only indirectly regulate lipogenesis genes by inducing SREBP-1c, ChREBP, and LXRα, or by crosstalk with insulin signaling. Due to unique regulatory mechanisms, the role of THR in lipogenesis is more tissue-specific and context-dependent, including nutritional and metabolic factors.

With simultaneously processing lipolysis, lipid beta-oxidation, mitochondrial thermogenesis, and lipogenesis in various mechanisms orchestrated by THR, brown adipocytes own a highly dynamic lipid turnover which is crucial to maintain energy homeostasis, metabolic balance, and fatty acid availability for the purpose of fuel supply, protein modification such as protein palmitoylation (Leishman et al., 2023; Yamamoto et al., 2024), and signaling molecules such as fatty acid as a ligand for PPARγ (Liberato et al., 2012).

The small size of lipid droplets in cells with THR overexpression in a glucose-rich environment reflects that THR promotes UCP-1 expression and preserves a dynamic lipid reservoir by highly activated lipid turnover for thermogenesis. However, fructose abolished this THR action on lipid turnover in brown adipocytes.

Cells respond to their surrounding environment, such as nutritional state, metabolic stress, proliferation, or differentiation stimuli, by secreting proteins as signals or responses for survival, protection, or adaptation. These secreted proteins are crucial for many pathogenesis processes of diseases (Heutling et al., 2004; Bradshaw, 2012; Kim et al., 2003). Proteins can be secreted into the media via the conventional ER-Golgi pathway, which is characterized by signal peptides attached to the proteins, or through non-conventional pathways, such as exosomes or macrovesicles (Deshmukh et al., 2019; Huberts and Van Der Klei, 2010; Nickel and Rabouille, 2009), particularly when cells are under stress. To provide independent, unbiased evidence to explore the mechanistic model that fructose impairs THR transcriptional competence, we performed proteomic analysis on the secretomes from differentiated brown adipocytes. The proteomic profiling data revealed that both the sugar source and THR overexpression have a profound impact on the secreted protein composition. These alterations in the secretome provide strong evidence that fructose dysfunctions THR transcriptional activity through oxidative and proteostatic stress. First, several fructose-induced secreted proteins we identified are functionally linked to oxidative stress, a process that modifies redox-sensitive cysteine residues and impairs nuclear receptor DNA-binding activity. For example, fructose markedly reduced the secretion of peroxiredoxin-6 (Prdx6), an antioxidant enzyme that detoxifies hydrogen peroxide and lipid hydroperoxides. Reduced Prdx6 secretion is consistent with an increased oxidative burden, which we propose may oxidatively modify the THR zinc-finger DNA-binding domain and contribute to impaired THR–TRE interactions. Second, fructose increased the secretion of proteins involved in protein folding and chaperone function (e.g. HSP90B, BiP). These pathways are known to regulate nuclear receptor stability and ligand responsiveness. Their upregulation suggests that fructose imposes proteostatic stress, potentially destabilizing THR or altering its transcriptional competence. Third, fructose increased secretion of proteins associated with glycolysis, the TCA cycle, and the malate–aspartate shuttle, consistent with metabolic rewiring that reduces mitochondrial oxidative capacity. Because THR-driven UCP1 expression requires intact mitochondrial function and lipid turnover, these metabolic shifts provide additional context for how fructose may blunt THR-dependent thermogenic programming. Finally, the THR overexpression itself reshaped the secretome, reducing proteins involved in glycolysis, RNA processing, and chaperone activity. These THR-dependent changes support the notion that THR activity is tightly coupled to cellular proteostasis and metabolic state, and that fructose disrupts this coupling.

It is important to emphasize that the current study was designed to specifically dissect the cell-autonomous mechanisms by which fructose affects brown adipocyte differentiation and thermogenic programming under well-controlled nutrient conditions. Using an established immortalized brown preadipocyte model, we identified a previously unrecognized mechanism by which fructose induces thyroid hormone resistance and impaired transcriptional activity, leading to suppression of UCP-1 expression.

Emerging *in vivo* studies support the physiological relevance of our findings. For example, in a rodent model fed a high-fat, high-fructose diet, BAT shows whitening phenotypes and reduced UCP1 protein levels, alongside decreased PGC1α and increased apoptotic signaling, indicating diminished thermogenic capacity in BAT due to both the fructose-containing diet and the fat content (Zwarts et al., 2019; Shree et al., 2024; Zhao et al., 2025). These studies directly link a fructose-rich diet to impaired BAT thermogenic markers in an *in vivo* rodent model. Future *in vivo* studies are needed to validate the effects of fructose on thyroid hormone signaling and BAT function. Our study provides important mechanistic insight that will guide and inform future *in vivo* investigations.

### Conclusions

Our data demonstrate that fructose reduces the number and size of cellular lipid droplets, downregulates brown adipogenesis markers, and the brown adipocyte identity marker UCP-1. Overall, fructose prevented brown adipogenesis. Elevating the THR ligand T3 and the THRβ agonist resmetirom does not rescue or increase UCP-1 expression in a fructose-rich environment, suggesting that fructose induces thyroid hormone resistance. Fructose downregulates THR through ubiquitin-mediated protein degradation, and resuming THR expression does not rescue UCP-1 expression, which provides further evidence to support the notion that fructose causes thyroid hormone resistance. Proteomic profiling of brown adipocyte secretomes revealed that both the sugar source and THR overexpression have a profound influence on the secreted protein composition. Fructose increased the secretion of proteins related to glycolysis, TCA cycle, mRNA processing, protein folding, and cell signaling, which is consistent with feedback regulation of energy metabolism under fructose carbon availability. Fructose decreased the proteins involved in redox defense, which may impair the functions of THR. Excess THR activity in a fructose environment decreased the proteins related to energy metabolism and protein chaperoning. This may increase the risk of protein misfolding and stress, which could also be the molecular mechanism of thyroid hormone resistance. These data indicate that fructose disrupts energy metabolism, redox protection, and protein folding, which may contribute to THR dysfunction and impair adipogenesis. Thus, fructose promotes thyroid hormone resistance to UCP-1 expression and impairs brown adipogenesis. Future investigations are needed to explore the defects and mechanisms underlying the disruption of THR regulation of UCP-1 and brown adipogenesis in a fructose-rich environment.

## MATERIALS AND METHODS

### Retroviral production

HEK-293T cells (ATCC, cat. no. CRL3216) served as producer cells. The contamination-free cell line was certified by ATCC and tested by our laboratory before use in the studies. The cells were seeded at 1.2×10^6^ cells/well in a six-well plate using 2 ml of packaging medium (Opti-MEM reduced serum medium with 5% FBS, (Thermo Fisher Scientific, cat. no. 31985070). The next day, the cells were transfected with a retroviral packaging vector (pCL-Eco, Addgene, plasmid no. 12371), an envelope protein vector (pVSV-G, Addgene, plasmid no. 138479), and an expression vector (pBabe-neolarge TcDNA, Addgene, plasmid no. 1780) using Lipofectamine 3000 transfection reagent (Thermo Fisher Scientific, cat. no. L3000001). The viral particles were collected at 24 h and 52 h post-transfection and then centrifuged and purified. The viral particles were quantified using a commercially available QuickTiter retrovirus quantification kit (Cell BioLabs, cat. no. VPK-120), and then aliquoted and stored at −80°C (Wu et al., 2024).

### Isolation, culture, and immortalization of mouse brown preadipocytes

Animal protocols were approved by the Institutional Animal Care and Use Committee (IACUC) of the University of Maryland School of Medicine and were in accordance with National Institutes of Health (NIH) guidelines. The C57BL/6J mice were from The Jackson Lab (cat. no. 000664). Only one animal was used as a sole donor of BAT. Brown preadipocyte isolation and immortalization were previously described with modifications (Wu et al., 2024; Shamsi and Tseng, 2017). Briefly, after being anesthetized with isoflurane, BAT from a 1-day-old male mouse pup was removed from the interscapular region, minced into small pieces, and digested in buffer including 1 mg/ml collagenase II (Sigma, cat. no. C6885) at 37°C for 30-40 min. digested tissues were then filtered through a 100 μm cell strainer into a new 50 ml sterile tube. Cells were then pelleted by centrifugation at 600 ***g*** for 5 min and plated into a six-well culture plate containing growth medium (DMEM/F12, 10% FBS, and 100 μg/ml primocin). The cells were cultured overnight at 37°C and 5% CO_2_. Cells were washed with pre-warmed 1x PBS and then infected with retrovirus to express SV40 large T antigen. After being infected for 48 h, the cells were cultured in selection medium (growth medium supplied with 450 μg/ml neomycin). When the cells reached 80-90% confluence, they were sub-cultured to a new six-well plate. Cells were maintained in neomycin selection for at least 7 days. Cells were tested to ensuring contamination free and characterized by cell morphology, lipid droplet morphology, and brown adipocyte markers before use in our studies (Wu et al., 2024).

### Differentiation of mouse primary brown preadipocytes

Immortalized preadipocytes were grown in the DMEM medium supplemented with 1 g/l glucose, 10% FBS, and 100 μg/ml primocin. When cells reached 100% confluence, differentiation (day 0) was initiated with the induction medium with 1 g/l glucose and/or 1 g/l fructose supplemented with 0.5 mM IBMX, 20 nM insulin, 125 nM indomethacin, 1 nM triiodothyronine (T3), 1 μM dexamethasone, and 1 μM rosiglitazone for 48 h. Cells were then incubated in maintenance medium supplemented with 1 g/l glucose and/or 1 g/l fructose, along with 20 nM insulin and 1 nM triiodothyronine (T3) for 12 days (Wu et al., 2024). The fructose concentration (1 g/l, or 5.5 mM) used in this study is commonly used *in vitro* and has been reported in numerous studies (Rella et al., 2025; Huang et al., 2011). This study is designed to model cell-autonomous responses to fructose exposure under control conditions and to interrogate the underlying mechanisms by which fructose can alter brown adipocyte differentiation, rather than to precisely mimic circulating concentrations.

### AAV-mediated overexpression of THR

Immortalized brown preadipocytes were seeded in 2 ml of cell growth medium supplemented with 1 g/l glucose and 10% FBS at a density of 1×10^5^ cells per well in a six-well plate. The next day, the first AAV transduction was performed. The cell culture medium was replaced with 1 ml of fresh growth medium. The volume of AAV for infection was calculated based on parameters (MOI: 10^4^-5×10^5^, Cell number: 1×10^5^, Titer of AAV-mTHR: 2.84×10^12^ GC/ml, Titer of AAV control: 7.82×10^11^ GC/ml). AAV volume for infection=(MOI×Cell number)/AAV Titer. A total of 20 μl of AAV for both the target THRβ and control was added to the cell culture medium. AAVs were from Vector Builder. The catalog number of AAV-mTHR is AAV8L (VB240513-1749wjm), and that of AAV-control is AAV8C(VB010000-9394npt)-b. After 24 h of infection, the medium containing AAV was removed and replaced with fresh growth medium. When cells reached 100% confluence, cell differentiation was initiated by adding the induction medium with 1 g/l glucose and/or 1 g/l fructose. After 48 h of induction, the second AAV transduction was performed. At 24 h post-transduction, cells were cultured in maintenance/differentiation medium. The medium was changed every day until day 12 of differentiation.

### Lipid droplet staining and nucleus counterstaining

Live cell lipid droplet staining was achieved by HCS LipidTOX deep red neutral lipid stain (Thermo Fisher Scientific) or BODIP™ 493/503 green stain (Thermo Fisher Scientific). Cell nuclei DNA was stained by Hoechst 33342 (Thermo Fisher Scientific). Lipid and nuclear staining medium were prepared by adding 5 μl of HSC lipid TOX deep red neutral lipid stain (1:200 dilution), or 0.4 μl of BODIPY stock solution (5 mM), and 1 μl of Hoechst 33342 working solution (1 mg/ml) to 1 ml of maintenance medium and mixing well. Lipids and nuclei were stained by adding a prepared staining medium to the cells (1 ml/well in a six-well plate). Cells were incubated in a cell culture incubator for 30 min and then imaged with a fluorescence microscope (Wu et al., 2024).

### Cell imaging and quantitative analysis

After incubation for lipid and nuclear staining, cells were immediately observed under a microscope. Cell images were acquired using Leica DMi8 microscope. Cell images under visible light were acquired using Flexacam C1 camera with phase contrast and color channel settings. Cell fluorescence images were acquired with a Leica K5 camera with multi-fluorescence channel settings. The magnification was 10×20. The wavelength of fluorescence excitation/emission is 637/655 nm for lipid TOX deep red neutral lipid stain, 493/503 nm for BODIPY green neutral lipid stain, and 460/360 nm for Hoechst 33342 nuclei stain. Cell images were processed by Leica Application Suite X software. Fluorescence images were analyzed using ImageJ-equivalent workflows in Python. Lipid droplets with red or green channels were separated, and automated thresholding was performed using Otsu's method. Binary masks were cleaned using size-based filtering to remove background noise. Individual lipid droplets were identified by connected-component labeling, and lipid droplet area was quantified in pixel units for each object.

### Western blot

On day 12 of differentiation, the cell culture medium was aspirated, and 400 μl of Roth lysis buffer (50 mM Hepes pH7.4, 150 mM NaCl, 1% NP-40, 5 mM EDTA, 5 mM EGTA, 20 mM Na Pyrophosphate, 20 mM NaF, 1 mM MgCl_2_, 10% glycerol, 0.5 mM DTT) with protease and phosphatase inhibitors (Thermo Fisher Scientific) was added to the cells in each well of the six-well plates. Cells were then collected into 1.5 ml tubes and sonicated for 10 s on ice. Cell lysate was centrifuged at 13,000 rpm for 10 min. The supernatant of the cell lysate was transferred into a new 1.5 ml tube. Protein concentration of the cell lysate was assayed using the RC DC protein assay kit (Bio-Rad). DNA concentration of the cell lysate was also measured by dsDNA quantification kits (Thermo Fisher Scientific) for reference. Each protein sample, with the same amount of protein, was mixed with Laemmli sample buffer containing 2-mercaptoethanol and then heated at 100°C for 5 min to denature the protein. The protein samples were loaded into a 4-20% Criterion TGX precast protein gel (Bio-Rad) for separation by polyacrylamide gel electrophoresis (SDS-PAGE) based on their size. Proteins were then electro-transferred from the gel to the nitrocellulose membrane. The membrane was incubated in blocking buffer (5% non-fat dry milk in 1× TBST) for 1 h at room temperature and then incubated with the primary antibody in blocking buffer overnight at 4°C. The membrane was washed twice in washing buffer (1× TBST) and then incubated in the secondary antibody conjugated with horseradish peroxidase (HRP) for 1 h at room temperature. After washing three times in washing buffer, the chemiluminescent substrate (Thermo Fisher Scientific) was applied to the membrane. The chemiluminescent signal was detected and imaged using Syngene G:BOX gel and blot imaging system. The blot bands were analyzed by ImageJ. All antibodies used in the studies were listed in Table S1.

### Co-immunoprecipitation

Cell lysate of a differentiated brown adipocyte preparation was described in the western blot section. The 500 μg total protein from each sample was incubated with 30 μl protein A/G magnetic beads (Thermo Fisher Scientific) at room temperature for 1 h to pre-clean the protein from nonspecific binding to the beads. After separating from precleaning beads, the protein samples were then incubated with 1.5 μg of THRα1β1 mouse monoclonal antibody (Santa Cruz Biotechnology) at 4°C overnight. The 30 μl protein A/G magnetic beads were added and incubated with the protein-antibody mixture at room temperature for 2 h. The beads with the antibody and captured proteins, separated from the protein sample, were washed three times with 1000 μl of 1×PBS each time. The proteins and their binding partners captured by the antibody and beads were eluted and separated by adding 45 μl of 1×Laemmli sample buffer (Bio-Rad) and heating at 100°C for 5 min. The captured proteins in 1×Laemmli sample buffer were then separated from the beads and loaded into a 4-20% Criterion TGX precast protein gel (Bio-Rad) for separation by polyacrylamide gel electrophoresis (SDS-PAGE). After being electro-transferred from the gel to the nitrocellulose membrane, the captured proteins were immunoblotted with a ubiquitin rabbit monoclonal antibody (Cell Signaling Technology) at a 1:1000 dilution overnight at 4°C. The membrane was washed twice in washing buffer (1×TBST) and then incubated in the secondary antibody conjugated with HRP for 1 h at room temperature. Chemiluminescent signal detection and imaging are described in the western blot section. Antibodies used for co-immunoprecipitation and western blot were listed in Table S1.

### Secretome analysis by mass spectrometry

Secretome analysis by LC-MS has been previously described with modifications (Deshmukh et al., 2019; Villarroya et al., 2019). Briefly, after incubating with cells for 24 h, the cell culture media, which served as maintenance media containing glucose or fructose, were collected on day 12 of brown adipocyte differentiation. Three independent replicates were prepared for each sample. Proteins from media were precipitated by MeOH/CHCl_3_ (Zhang et al., 2019) and then digested with porcine trypsin (Promega). Pooled peptides were cleaned up and analyzed by liquid chromatography (nanoAcquity, Waters) coupled to a mass spectrometer (LTQ-Orbitrap Velos, Thermo Fisher Scientific). We performed a targeted over-representation analysis. We first inspected the recurring proteins across conditions and grouped them into coherent functional modules such as glycolysis/TCA metabolism, protein folding, RNA processing, chromatin, signaling hubs, growth factor transport, and innate immunity. Each module was then mapped to its canonical Gene Ontology Biological Process term. This restricted set of Gene Ontology terms was used for the hypergeometric enrichment test, thereby limiting the multiple-testing burden and allowing for focused biological interpretation. The proteomic dataset is intended as a supporting mechanistic dataset that complements our investigation on fructose-induced THR dysfunction, not as a standalone resource.

### Statistical analysis

The different groups of cells were cultured in identical conditions except for the designed treatments. Based on preliminary data, the expected difference between groups was approximately 50%, with a standard deviation of 15%. A power calculation (α=0.05, power=0.80) indicated that *n*=3 biological replicates per group was sufficient. Data with normal distribution are reported as means±s.e.m. and analyzed with GraphPad Prism 9 software (GraphPad Software, Boston, MA, USA). We used one-way ANOVA followed by a post hoc Tukey test for multiple comparisons. Statistical significance is indicated as *P*<0.05. Columns labeled with different lowercase letters are statistically different by ANOVA. Violin plot, volcano plot, and heatmap were generated by Python.

## Supplementary Material

10.1242/biolopen.062648_sup1 Supplementary information
