# Supplementary material for: Fructose impairs brown adipogenesis by promoting thyroid hormone resistance in differentiated brown adipocytes
Source: Biol Open. 2026 Jun 22;15(6):bio062648. doi: 10.1242/bio.062648 (PMC13382702; doi:10.1242/bio.062648)
Supplement: Supplementary information [file biolopen-15-062648-s1.pdf]

Table S1. Antibodies for Western blot and Co-immunoprecipitation

Target Protein	Supplier / Catalog #	Description / Clone # / Lot #	Validation Reference
PPARγ	Cell Signaling Cat. # 2443	PPARγ (81B8) rabbit monoclonal antibody<br>Lot # 6	Chien et al., 2026. Hepatol Commun. PMC13004210
UCP-1	Cell Signaling Cat. #14670	UCP-1 (D9D6X) rabbit monoclonal antibody<br>Lot # 2	Xiao et al., 2026. PMC12987616
FABP4	Cell Signaling Cat. # 2120	FABP4 rabbit polyclonal antibody<br>Lot # 3	Wang et al., 2025. Nat Commun. PMC12635226
Ubiquitin	Cell Signaling Cat. # 20326	Ubiquitin (E6K4Y) rabbit monoclonal antibody<br>Lot # 3	Monti et al., 2026. Cell Death Dis. PMC13039404
THRα1/β1	Sant Cruz # sc-739	THRα1/β1 (C1) mouse monoclonal IgG1k antibody<br>Lot # B2315	Guan et al., 2017. PNAS. PMC5547603
RXRα/β/γ	Sant Cruz # sc-46659	RXRα/β/γ (F1) mouse monoclonal IgG1k antibody<br>Lot # C3022S	Chen et al., 2024. Heliyon. PMC11261804
Vinculin	Cell Signaling Cat. # 13901	Vinculin (E1E9V) rabbit monoclonal antibody<br>Lot # 10	Liu et al., 2026. Nat Cell Biol. PMC7618962
β-actin	Cell Signaling Cat. # 20326	B-actin (8H10D10) mouse monoclonal IgG2b antibody<br>Lot # 23	Niu et al., 2026. Genes Dis. PMC12914543

Biology Open • Supplementary information
